# Maternal fatigue in postpartum and adjustment to motherhood: an exploratory study

**DOI:** 10.1192/j.eurpsy.2026.11943

**Published:** 2026-07-31

**Authors:** I. M. M. M. D. Mendes, S. M. Coelho, A.-B. Caetano, C. Castelo-Branco, R. M. C. Rodrigues

**Affiliations:** Nursing School of Coimbra, Coimbra, Portugal

## Abstract

**Introduction:**

The birth of a child is a challenging event that requires constant adjustment to parenthood for both parents. Postpartum fatigue is a decrease in physical and mental capacity, lack of energy, and decreased concentration (Henderson et al., 2019). It affects both parents and consequently affects their routines. Postpartum fatigue can be associated with depression, changes in sleep patterns, breastfeeding difficulties, and problems bonding with the baby.

**Objectives:**

To describe maternal fatigue up to six months postpartum; identify maternal needs regarding fatigue up to six months postpartum.

**Methods:**

Exploratory-descriptive quantitative study, using a non-probability snowball sample of 120 postpartum women, after obtaining informed consent. The questionnaire consisted of sociodemographic and obstetric questions and the Portuguese version of the abbreviated Fatigue Symptom Checklist (FSC) for women and postpartum fatigue. Data collection conducted through an online questionnaire after the study received ethical approval from the Ethics Committee of the Research Unit. Data analyzed using SPSS, version 27.

**Results:**

The main results of the study highlight that motherhood is a challenging and tiring experience (95.8%), 34.2% of the participating mothers do not ask for help when they feel fatigued, and 36.7% ask their partner for help. First-time mothers experience higher levels of postpartum fatigue due to adjusting to a new reality, insecurity regarding baby care, and more difficulties in postpartum recovery with lower energy levels (96%). It is noteworthy that 65% of mothers participated in a childbirth preparation program, and of these, 45.8% reported that the topic of postpartum fatigue did not approached.

**Conclusions:**

This exploratory study highlights the importance of monitoring mothers, identifying the factors that can lead to maternal fatigue and developing support strategies for a healthy adjustment to motherhood, helping to deal with difficulties and deviations caused by maternal fatigue, developing interventions to, equally, involve the family in the transition to parenthood in a healthy way.

**Disclosure of Interest:**

None Declared

